# Intervention study of tai chi training on the intestinal flora of college student basketball players

**DOI:** 10.1097/MD.0000000000035044

**Published:** 2023-09-08

**Authors:** Dongyang Kang, Xiaorong Wang, Jiahong Wang

**Affiliations:** a Chengdu Sport University, Chengdu, Sichuan Province, China; b Department of Physical Education, Henan University of Science and Technology, Luoyang, China; c Department of Physical Education, Luoyang Polytechnic, Luoyang, China; d Department of Physical Education, Soochow University, Suzhou, China.

**Keywords:** 16S rRNA, college basketball player, health promotion, intestinal flora, tai chi training

## Abstract

**Background:**

In recent years, the interactions between host and host-associated gut flora have received increasingly widespread attention. Tai chi is a traditional Chinese exercise, which can significantly benefit adults with health condition. But the studies on the function of intestinal flora and its correlation with tai chi exercise, are limited. In addition, the influence of tai chi on intestinal flora has largely been understudied. In this study, we investigated the changes in intestinal microflora by 16S rRNA sequencing to clarify the specific mechanism of tai chi on the regulation of intestinal flora and seek to formulate a reasonable “exercise prescription.”

**Methods:**

**Design:** randomized controlled trial. **Participants:** thirty college basketball players randomly divided into control (n = 15) and test (n = 15) groups. **Intervention:** experimental group practiced 24-style simplified tai chi ≥ 6 times/week for 20 weeks. **Outcomes:** serum biochemical markers, blood pressure, and intestinal microbial composition measured post-intervention. **Analysis:** intent-to-treat analysis.

**Results:**

**Primary outcomes:** after tai chi intervention, the level of high-density lipoprotein cholesterol (1.22 mmol/L) and triglycerides (0.64 mmol/L) were significantly decreased. **Secondary outcomes:** aspartate transaminase, alanine aminotransferase, total cholesterol, and low-density lipoprotein remained unchanged. **Microbiota:** α-diversity index significantly increased, particularly with increased Blautia. **Blood pressure:** test group showed significantly lower diastolic blood pressure (83–95 mm Hg) compared to control.

**Discussion:**

Considering other markers, increased gut flora diversity during exercise may imply a healthier gut environment. Physical exercise promotes a decrease in the inflammatory process by reducing the levels of bacteria associated with pro-inflammation, such as Proteobacteria. Further research is required to understand the nuanced link between gut flora diversity and exercise intensity.

**Conclusion:**

24-style simplified tai chi enhances human intestinal flora diversity. Improvements observed in blood lipid profiles and blood pressure levels.

## 1. Introduction

With the development of social economy and the progress of science and technology, changes in human behavior not only have affected the ecological environment, but also the human body’s internal microbial environment. The microbiota may be considered the proximate environmental factor conferring risk or resistance to a range of chronic inflammatory and metabolic disorders, which are common in socio-economically developed societies.^[1]^

The human gut is colonized by microorganisms that are comparable in number to its own cells^[2]^; these microorganisms can encode a wide variety of enzymes that are closely related nutrition, metabolism, and immunity^[3,4]^ of the host and are influenced by a variety of factors, such as the host’s genetic background, age, health status, diet, drug use, and lifestyle.^[5,6]^

Under normal conditions, various microorganisms maintain symbiotic or antagonistic relationships in the gut and together form a dynamically balanced microecosystem. In recent years, the interactions between host and host-associated gut flora has received increasingly widespread attention. Healthy intestinal flora has functions, such as protecting the intestine, improving metabolism, and regulating body immunity, anti-inflammation, and anti-tumor activities, and plays an important role in the development of numerous diseases. Most of the elements of the modern society have a modifying effect on the intestinal flora, but one that has received comparatively little attention is exercise. One way to effectively promote the health of the body and improve the quality of life is through proper and appropriate exercise, which is important in suppressing the inflammatory response and promoting immune regulation of the body.^[7]^ Clarke SF et al showed that exercise optimizes the structure of the host intestinal flora and improves intestinal microecology.^[8]^

Tai Chi is a traditional Chinese sport. It is a physical and mental exercise that uses breath-controlled continuous, bending, and spiral body movements, which can improve a person’s aerobic capacity, muscle strength, balance, and motor control, reduce stress and anxiety, and improve the quality of life.^[9,10]^ Traditional tai chi effectively mobilizes multiple organs throughout the body, and to a large extent regulates and improves the function of the cardiovascular, digestive, and circulatory systems.^[11,12]^

At present, “high” and “moderate” quality evidence indicates that tai chi can significantly benefit adults with health conditions including cancers, chronic obstructive pulmonary disease, coronary heart disease, depression, heart failure, hypertension, low back pain, osteoarthritis, osteoporosis, Parkinson disease, and stroke.^[13]^ However, although studies have confirmed the regulatory and optimization effects of exercise on intestinal flora, the studies on the function of intestinal flora and its correlation with tai chi exercise, are limited. In addition the influence of tai chi on intestinal flora has largely been understudied. In this paper, we investigated the changes in intestinal microflora by 16S rRNA sequencing to clarify the specific mechanism of tai chi on the regulation of intestinal flora and seek to formulate a reasonable “exercise prescription.”

## 2. Research methodology

This study is a randomized controlled trial. The research scheme of this research project has been approved by the ethics committee of Henan University of Science and Technology (Ethics record number:2022-03-B109) and is in line with the provisions of the Helsinki declaration. All subjects provided written informed consent.

### 2.1. Participants

A total of 30 student basketball players from a university in Henan were selected and randomly divided into a control (15) and an experimental group (SPT) of 24-style simplified tai chi (15). No significant differences in age were observed in the age, height, and weight of the subjects between the 2 groups before the experiment. All subjects were screened by medical history and physical examination, free of autoimmune diseases, serious mental diseases, tumors, and endocrine abnormalities, and allowed to exercise at moderate intensity after exercise risk assessment (60–70% of maximum heart rate).

In addition, all subjects received a diet and physical activity questionnaire before the experiment began, and no significant differences were observed in their daily diet and physical activities. The diet consisted of rice, noodles, vegetables, and several meats; physical activities comprised a physical education program and sports training activities within the basketball team training program. The participants reported no use of antibiotics, gastrointestinal drugs, micro-ecological agents, and other agents affecting intestinal flora, etc; no history of diarrhea, dysentery, and other gastrointestinal diseases; no previous history of gastrointestinal surgery within the past 1 month.

### 2.2. Study design

The experimental and control groups (CTRLs) kept their original routine unchanged. The SPT practiced 24-style simplified tai chi under the guidance of a tai chi instructor every day at 18:30 in the martial arts hall. The group practiced 6 days a week for 1 hour and 30 minutes a day, including 7 to 8 times of 24-style simplified tai chi with music accompaniment, for about 1 hour and 15 minutes, and 15 minutes of preparatory activities and finishing exercises. The experimental period was 5 months. During the experiment, the subjects were asked to maintain the same lifestyle and diet, avoid adverse mental stimulation, and inhibit from taking microbiological agents.

The intensity of 24-style simplified tai chi practice was controlled by monitoring the heart rate during exercise with the “Black Plus” exercise bracelet, and the target heart rate reached 70% of the maximum heart rate during exercise.

### 2.3. Collection and analysis of serum biochemical indicators

For all basketball players, blood samples were collected from the elbow vein in the morning following an overnight fasting (>8 hours) after the test, and blood pressure was measured. Blood samples were allowed to stand at room temperature for about 30 minutes and then centrifuged at 2000 r/min for 8 minutes at 4 °C. The supernatant was separated and stored at −80 °C. Aspartate transaminase (AST), alanine aminotransferase (ALT), BG, triglyceride (TG), total cholesterol (TC), high-density lipoprotein (HDL) cholesterol, and low-density lipoprotein cholesterol were measured using a Myriad BS-400 automatic biochemical analyzer and the corresponding assay kits (BIOSINO Biotech Co., Ltd., China).

### 2.4. Stool sample collection and DNA extraction

#### 2.4.1. Sample collection.

Upon completion of the experiment, fresh fecal samples were collected from each athlete and immediately frozen in a −80 °C refrigerator.

#### 2.4.2. DNA extraction and polymerase chain reaction (PCR) amplification.

Total genome DNA from samples was extracted using the cetyltrimethylammonium bromide method. DNA concentration and purity was monitored on 1% agarose gels. Base on the concentration, DNA was diluted to 1 ng/µL using sterile water.

The 16S rRNA genes of distinct regions (16S V3_V4) were amplified using specific primers (341F (5′-CCTAYGGGRBGCASCAG-3′) and 806R (5′-GGACTACNNGGGTATCTAAT-3′)) with their barcodes. All PCR reactions were carried out with 15 µL Phusion® High-Fidelity PCR Master Mix (New England Biolabs), 2 µM forward and reverse primers, and 10 ng template DNA. Thermal cycling consisted of initial denaturation at 98 °C for 1 minute, followed by 30 cycles of denaturation at 98 °C for 10 seconds, annealing at 50 °C for 30 seconds, elongation at 72 °C for 30 seconds, and finally, 72 °C for 5 minutes

The same volume of 1×TAE buffer was mixed with the PCR products, and electrophoresis was conducted on 2% agarose gel for detection. The PCR products were mixed in equidensity ratios. Then, the mixture PCR products were purified with a Qiagen Gel Extraction Kit (Qiagen, Germany).

#### 2.4.3. Illumina NovaSeq sequencing.

Sequencing libraries were generated using TruSeq® DNA PCR-Free Sample Preparation Kit (Illumina, San Diego, CA) following manufacturer’s recommendations and index codes were added. The library quality was assessed on the Qubit@ 2.0 Fluorometer (Thermo Scientific, Waltham, MA). Finally, the library was sequenced on an Illumina NovaSeq platform and 250 bp paired-end reads were generated.

#### 2.4.4. Bioinformatics analysis.

The analysis was conducted by following the “Atacama soil microbiome tutorial” of Qiime2docs along with customized program scripts (https://docs.qiime2.org/2019.1/). Briefly, raw data FASTQ files were imported into a format which can be operated by QIIME2 system using the qiime tools import program.^[14]^ Demultiplexed sequences from each sample were quality filtered and trimmed, de-noised, and merged, and the chimeric sequences were identified and removed using the QIIME2 dada2 plugin to obtain the feature table of amplicon sequence variants.^[15]^ The QIIME2 feature-classifier plugin was then used to align amplicon sequence variant sequences to a pre-trained GREENGENES 13_8 99% database (trimmed to the V3–V4 region bound by the 338F/806R primer pair) to generate the taxonomy table.^[16]^ Any contaminating mitochondrial and chloroplast sequences were filtered using the QIIME2 feature-table plugin. Appropriate methods^[17]^ including the analysis of composition of microbiomes, analysis of variance (ANOVA), Kruskal–Wallis test, linear discriminant analysis effect size (LEfSe) and DEseq2, were employed to identify the bacteria with different abundances among samples and groups.^[18,19]^ Diversity metrics were calculated using the core-diversity plugin within QIIME2. Feature level alpha diversity indices, such as observed operational taxonomic units (OTUs), Chao1 richness estimator, Shannon diversity index, and Faith phylogenetics diversity index were calculated to estimate the microbial diversity within an individual sample. Beta diversity distance measurements, including Bray Curtis, unweighted UniFrac and weighted UniFrac measurements, were performed to investigate the structural variation of microbial communities across samples, and then the results were visualized via principal coordinate analysis and nonmetric multidimensional scaling.^[20]^ Unless specified above, the parameters used in the analysis were set as default.

### 2.5. Statistical analysis

Statistical analysis was conducted mainly in GrapPad Prism (version v6.01) and R4.2 software. Student *t* test was used to analyze the differences in physiological and biochemical indices between the test and CTRLs.

## 3. Results

### 3.1. Effect of tai chi on physiological and biochemical indexes of basketball players

**Figure 1.**
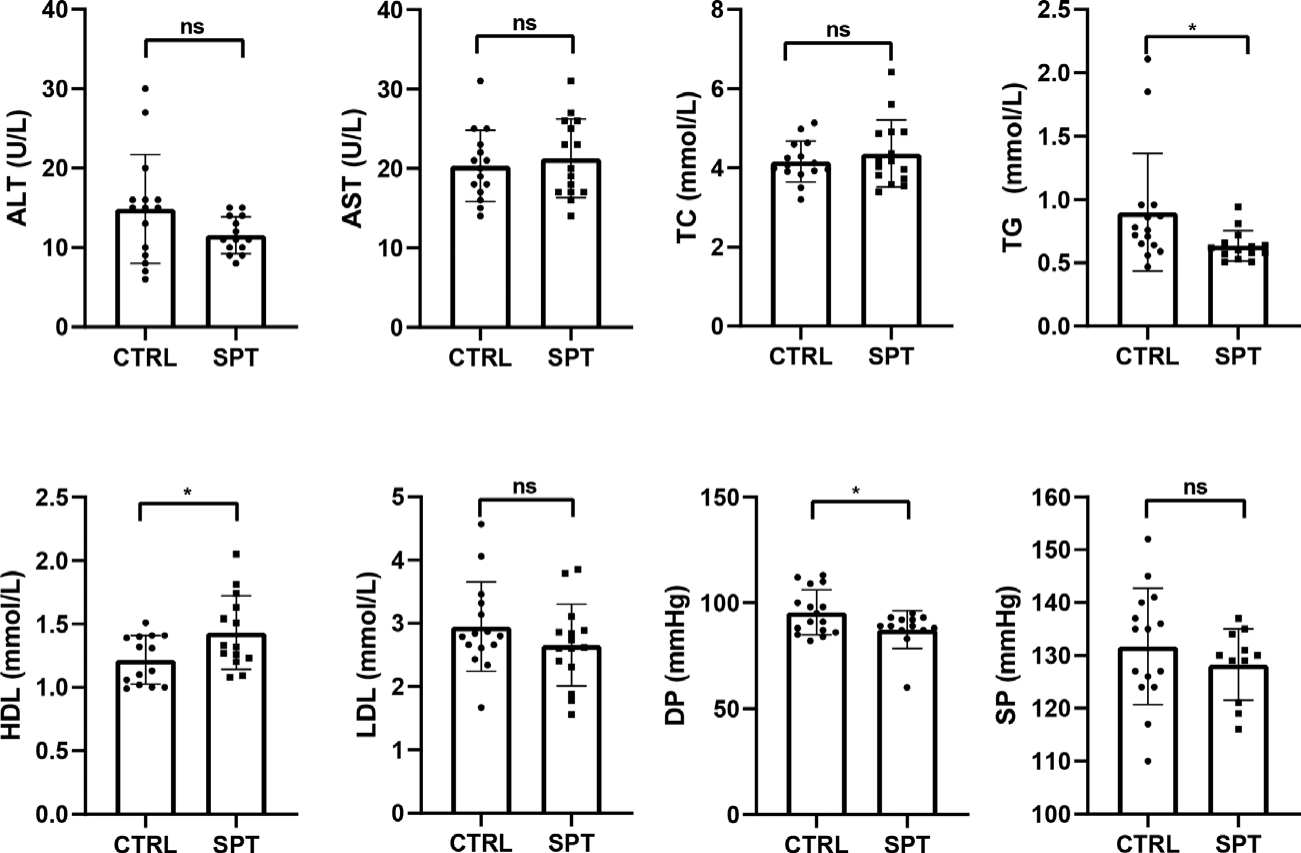
Effect of tai chi on physiological and biochemical indices.

As shown in Figure 1, no significant difference was observed in the ALT, AST, TC, low-density lipoprotein, and systolic pressure in the test group (tai chi intervention group) compared with the CTRL, whereas the TG, HDL, and blood pressure levels decreased significantly with tai chi intervention (*P* < .05).

### 3.2. Analysis of the effect of tai chi on the alpha diversity of intestinal flora

**Figure 2.**
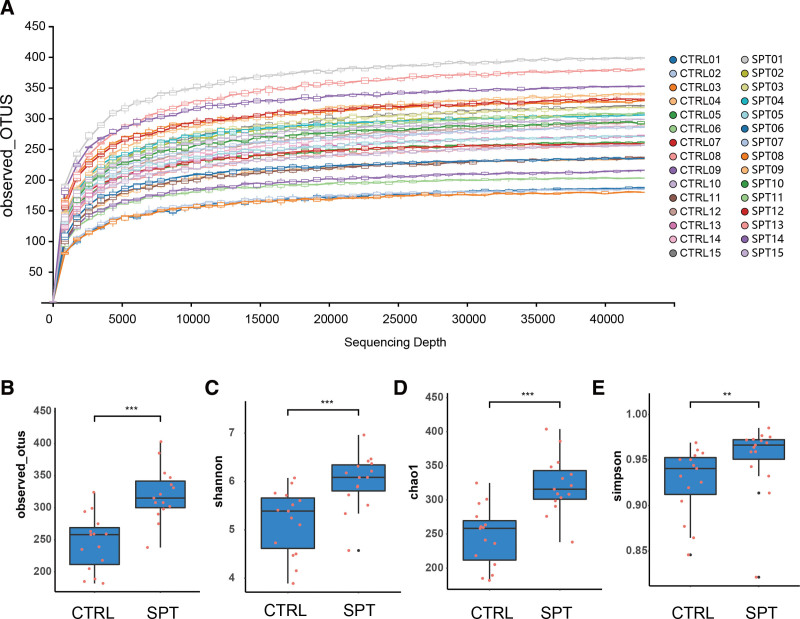
Alpha-diversity of gut flora in basketball players with tai chi intervention; (A) rarefaction curve analysis of stool samples based on observed_otus; (B–E) represent CTRL vs SPT for observed_otus, shannon, chao1, simpson box plots, respectively; “*” represents *P* < .05, “**” represents *P* < .01, “***” represents *P* < .001.

Accurate determination of microbial communities requires high sequence extraction and sequencing depth. The sparsity curve can be used to explore the trend of sample alpha diversity with the extraction leveling depth to determine, to a certain extent, whether the sequencing data have reached saturation. Figure 2A shows the OTU-based sparsity curve of the bacterial population. The dilution curve entered a plateau when the sampling depth reaches 10,000, indicating that the sequencing results were sufficient to reflect the diversity contained in the current samples, and that the results are reliable.

To evaluate the alpha diversity of the microbial community, we used the Observed_otus index to characterize the richness of the microbial community, and the higher the value of observed species index, the higher the richness of the microbial community; the higher the value of Shannon and Simpson indexes, the higher the diversity of the microbial community. The results showed that compared with the CTRL, the observed species, Shannon, Simpson and Chao1 indexes of the test group were significantly higher (Fig. 2B–E), indicating that the tai chi intervention helped to maintaining the stability and diversity of the university basketball players’ flora.

### 3.3. Analysis of the difference of tai chi on the structure of intestinal flora of basketball players

**Figure 3.**
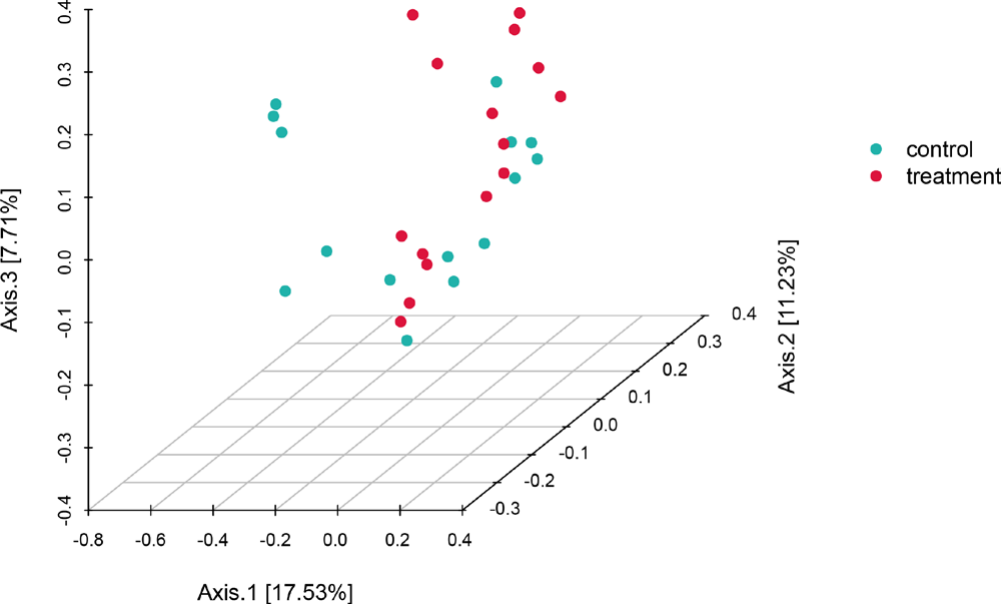
Principal coordinate analysis based on Bray-Curtis distance.

The principal coordinate analysis based on Bray_Curtis distance was performed on the intestinal flora of the test and CTRLs. Principal component (PC) 2 = 11.23%, PC3 = 7.71% were selected for graphical presentation (Fig. 3). The horizontal coordinates represent one PC, the vertical coordinates denote another PC, and the percentages indicate the contribution of the PCs to the variance of the samples. Each point in the graph represents a sample, and the samples of the same group are represented by the same color. The closer the samples are to each other, the more similar their species composition are. Thus, the samples with a high similarity in the community structure clustered together, and those with high a community variation were located distantly. As shown in the figure, the samples between the test and CTRLs were divided into 2 clusters, indicating that the OTU composition of the intestinal flora of the 2 groups of volunteers differed to a large extent and the structure of the intestinal flora differed significantly. Therefore, the distribution characteristics of the intestinal flora of the tai chi intervention exercise had significant individual differences, and the intensity of exercise can change the structure of the intestinal flora to certain extent.

### 3.4. Analysis of the difference in the composition of intestinal flora

**Figure 4.**
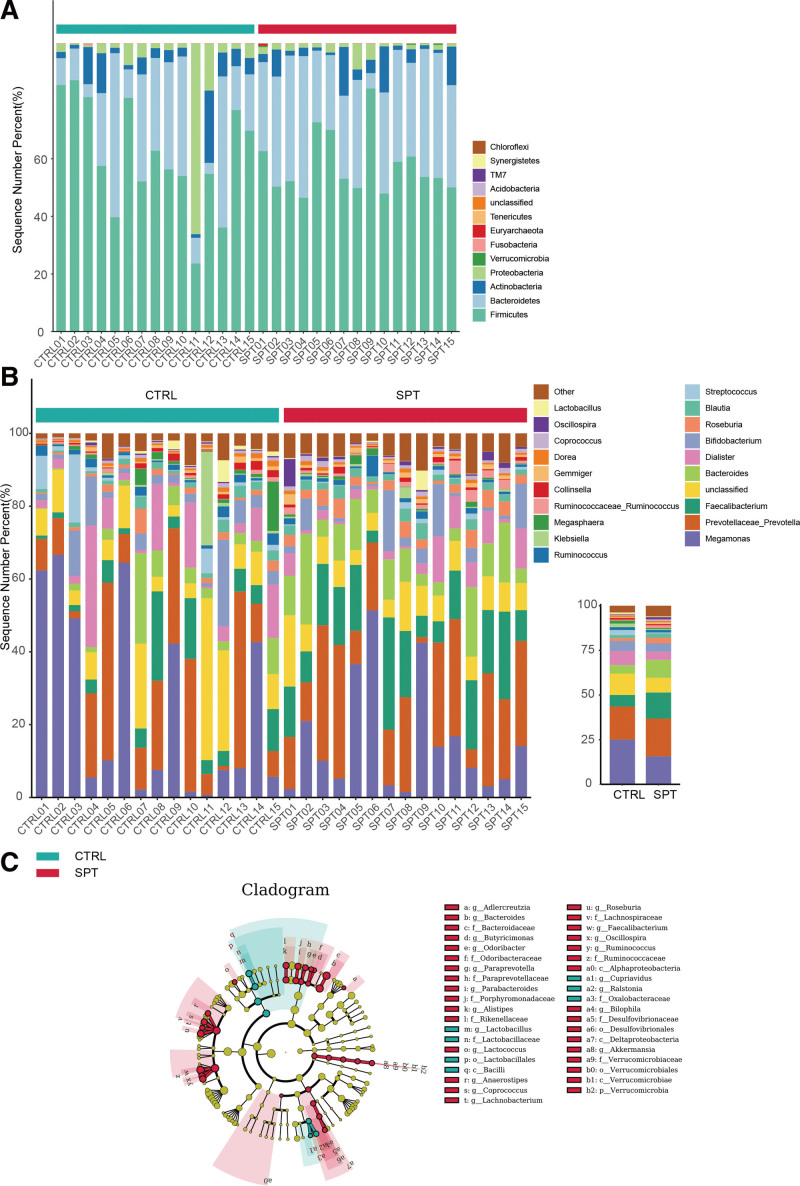
Intestinal flora composition and variance analysis; (A) phylum level composition; (B) genus level composition; (C) LEFSe analysis.

The results showed that the fecal flora of 30 basketball players mainly consisted of 4 phyla, Firmicutes, Bacteroidetes, Proteobacteria, and Actinobacteria, at the phylum level (Fig. 4). The abundance of Bacteroidetes in the intestinal flora of the tai chi intervention group increased significantly, whereas that of Bacteroidetes in the intestinal flora of the tai chi intervention group increased significantly. Meanwhile, the abundance of Proteobacteria decreased.

At the genus level, 19 genera with relative abundances above 0.5% were observed (Fig. 4B). The results of LEfSe analysis of samples from the 30 players showed significantly different species between the test and CTRLs at the family and genus levels (Fig. 4C). Ruminococcaceae, Lachnospiraceae, Rikenellaceae, and Paraprevotellaceae were significantly more abundant in the test group than in the CTRL, and Oxalobacteraceae, and Lactobacillaceae were significantly less abundant than those in the CTRL. Compared with the CTRL, the test group included significantly higher levels of Prevotella (21.14%), Faecalibacterium (14.63%), Bacteroides (10.00%), Roseburia (3.14%), Blautia (2.35%), Ruminococcaceae_Ruminococcus (1.48%), Gemmiger (1.02%), Coprococcus (0.95%), and Oscillospira (1.17%). Certain intestinal bacteria whose abundance was significantly increased in the tai chi intervention group can produce beneficial metabolites. An example is Prevotella, a bacterium commonly associated with a healthy plant-based diet, plays a beneficial role in the human body and is negatively associated with the development of diseases such as autism and Parkinson disease. Therefore, tai chi exercise can positively affect human health by increasing the levels of healthy bacteria and suppressing harmful bacteria through restructuring of the intestinal flora.

### 3.5. Correlation analysis of intestinal flora and physiological and biochemical indexes

**Figure 5.**
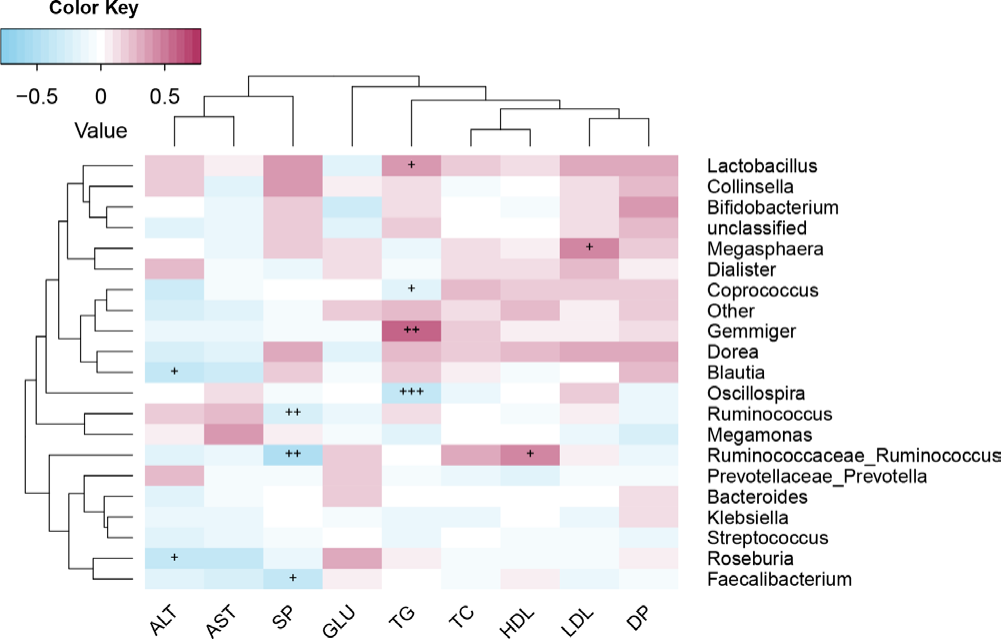
Correlation analysis of intestinal flora and serum biochemical indexes.

Tai chi intervention altered the structure/composition of the gut microbiota of basketball players and affected the changes in serum biochemical indicators (Fig. 5). The results showed that the microbiota Roseburia, Bacteroides, and Blautia were negatively correlated with ALT, and AST, whereas Ruminococcaceae_Ruminococcus was significantly positively correlated with HDL (*R* = 0.77, *P* < .05) and negatively correlated with SP. Oscillospira and Coprococcus showed a significantly negative correlation with TG, which proved that tai chi intervention can improve host health by affecting the intestinal flora.

## 4. Discussion

Exercise, as an environmental stimulus that can effectively promote the health of organisms, can regulate the structure of the host intestinal flora,^[21]^ and its effects on organisms (especially the gastrointestinal tract) are influenced by various factors, such as the exercise mode, intensity, and duration, suggesting that changes in the intestinal flora of the athletes might have been induced by variations in the exercise mode and conditions used in this study.

This study aimed to investigate the changes in intestinal flora diversity indices, OTU composition of the intestinal flora, fecal flora at the phyla level, LEfSe analysis result at the family and genus levels, and biochemical indexes in the 2 groups of college basketball players after a 20-week tai chi training intervention.

The effect of the tai chi intervention on the intestinal flora of the athletes was analyzed. The results obtained suggest that this structured training induces partial changes in the relative abundance and community structure of the intestinal flora. The species richness (OTU count) of the intestinal flora was significantly higher than that in controls. Considering other markers, Clarke et al observed that diabolic rugby players had significantly higher intestinal flora diversity than healthy individuals who were not athletes.^[8]^ Increased gut flora diversity during exercise may imply a healthier gut environment. However, the relationship between gut flora diversity and exercise intensity still needs further study. In addition, this study showed that the level of Proteobacteria decreased in the intestinal flora of the tai chi intervention group. Sabrina et al reported that after 9 weeks of indoor cycling training, the abundance of Proteobacteria decreased in the test population,^[22]^ suggesting the positive effect of exercise on promoting the host’s health. Therefore, physical exercise promotes a decrease in the inflammatory process by reducing the levels of bacteria associated with pro-inflammation, such as Proteobacteria.

In this study, we found tai chi intervention had a significant effect on the intestinal flora of the athletes, and the fecal flora was enriched with several butyrate-producing bacteria. Most of these were bacteria of phylum Firmicutes, such as Blautia and Roseburia. Butyrate is a major short-chain fatty acids considered a health-promoting molecule due to its role in the regulation of energy metabolism and increased insulin sensitivity. It can provide energy to intestinal epithelial cells, improve intestinal barrier function and reduce inflammatory responses, which are essential for maintaining intestinal health^[23]^ and protecting the gastrointestinal tract from injury caused by high-intensity training.

Given that tai chi is a standard aerobic exercise, various organs, and muscles are involved in the exercise process to consume oxygen and supply energy. Therefore, the body’s ability to take up and consume oxygen for energy is closely related to the diversity of the intestinal flora and butyrate production. Both factors are associated with overall host health.

## 5. Conclusion

In this study, by studying the effects of 24 styles of simplified tai chi on the intestinal flora of college basketball players, we observed that the intestinal flora diversity increased significantly, the abundance of harmful bacteria, such as Desulfovibrio spp. decreased significantly, and the TC level decreased significantly in the SPT, and the overall analysis showed that practicing 24 styles of simplified tai chi was beneficial to the health of the athletes.

## Author contributions

**Conceptualization:** Jiahong Wang.

**Data curation:** Dongyang Kang.

**Formal analysis:** Dongyang Kang.

**Investigation:** Dongyang Kang, Xiaorong Wang.

**Methodology:** Dongyang Kang.

**Project administration:** Jiahong Wang.

**Resources:** Jiahong Wang.

**Supervision:** Xiaorong Wang.

**Visualization:** Dongyang Kang.

**Writing – original draft:** Dongyang Kang.

**Writing – review & editing:** Dongyang Kang.
